# Use of residential wood heating in a context of climate change: a population survey in Québec (Canada)

**DOI:** 10.1186/1471-2458-8-184

**Published:** 2008-05-28

**Authors:** Diane Bélanger, Pierre Gosselin, Pierre Valois, Belkacem Abdous

**Affiliations:** 1 Institut national de santé publique du Québec and Centre de recherche du CHUQ, 945 Avenue Wolfe, Québec (Québec), G1V 5B3, Canada; 2 Institut national de santé publique du Québec, Ouranos and Université Laval, 945 Avenue Wolfe, Québec (Québec), G1V 5B3, Canada; 3 Faculté des Sciences de l'éducation, Université Laval, Québec (Québec), G1K 7P4, Canada; 4 Centre de recherche du CHUQ, 2875 boulevard Laurier, Édifice Delta 2, Québec (Québec), G1V 2M2, Canada

## Abstract

**Background:**

Wood heating is recommended in several countries as a climate change (CC) adaptation measure, mainly to increase the autonomy of households during power outages due to extreme climatic events. The aim of this study was to examine various perceptions and individual characteristics associated with wood heating through a survey about CC adaptations.

**Methods:**

A telephone survey (n = 2,545) of adults living in the southern part of the province of Québec (Canada) was conducted in the early fall season of 2005. The questionnaire used closed questions and measured the respondents' beliefs and current adaptations about CC. Calibration weighting was used to adjust the data analysis for the respondent's age and language under stratified sampling based on health regions.

**Results:**

More than three out of four respondents had access to a single source of energy at home, which was mainly electricity; 22.2% combined two sources or more; 18.5% heated with wood occasionally or daily during the winter. The prevalence of wood heating was higher in the peripheral regions than in the more urban regions, where there was a higher proportion of respondents living in apartments. The prevalence was also higher with participants completely disagreeing (38.5%) with the eventual prohibition of wood heating when there is smog in winter, compared to respondents somewhat disagreeing (24.2%) or agreeing (somewhat: 17.5%; completely: 10.4%) with the adoption of this strategy. It appears that the perception of living in a region susceptible to winter smog, smog warnings in the media, or the belief in the human contribution to CC, did not influence significantly wood heating practices.

**Conclusion:**

Increased residential wood heating could very well become a maladaptation to climate change, given its known consequences on winter smog and respiratory health. It would thus be appropriate to implement a long-term national program on improved and controlled residential wood heating. This would constitute a "no-regrets" adaptation to climate change, while reducing air pollution and its associated health impacts.

## Background

In Canada, minimum and maximum temperatures have increased over the last few decades, particularly in winter [1]. For instance, in southern Québec (south of the 49th parallel) average temperatures have increased by 0.5°C to 1.2°C based on an east-west trajectory [2]. These increases however, do not mean that climate warming is linear [3]. In fact, periods of intense cooling and severe storms are still predicted to occur. People will have to adapt appropriately, if only to prevent the health impacts of concern due to the cold [4,5].

A winter adaptation strategy is the use of residential wood heating. In Canada, more than 3 million dwellings use it as the primary or secondary source of heat [6]. Furthermore, the popularity of this type of heating grew in Québec to the point that its penetration rate increased by approximately 60% between 1987 and 2000 while the number of dwellings increased by less than 20% [7]. The massive and prolonged power outages that occurred in the middle of winter during the ice storm of 1998 [8] are thought to have played a significant role in this increase. In fact, the Web site of the Department of Natural Resources of Canada highlights this extreme climatic event in referring to evolved wood burning techniques as a means of coping with the worst winter storms [9].

Residential wood heating is also one of the main causes of winter smog in Canada. This type of heating is in fact responsible for 29% of Canadian emissions of fine particulates – one of the two key components of smog and its main winter component [10] from anthropogenic sources [11]. This relative contribution of fine particulates to emissions is even higher in Quebec at 47% [12] compared to most jurisdictions using fossil fuels for power generation, because hydroelectricity accounts for 96% of electricity production in the province and a great many homes use electric heat as the main source of heating.

Moreover, among the approximately one hundred atmospheric pollutants in wood smoke, several are greenhouse gases, while others are precursors of tropospheric ozone – the other key component of smog [13]. Furthermore, human exposure to fine particulates and tropospheric ozone is of particular concern because there are still no established concentration thresholds below which these pollutants are known to be safe and not pose a human health risk [13]. Young children, the elderly and people with respiratory problems (e.g., asthmatics) or heart problems are the most vulnerable, while healthy people who are repeatedly exposed, such as users of combustion units and their neighbours may also be at risk [7,14,15]. Finally, a well-known relationship exists between the harmful effects of these atmospheric pollutants and the increase in the number of visits to emergency rooms, hospitalizations, health care costs, absenteeism, the reduction in the labour force participation rate, as well as premature death [16].

Clearly, the rise in popularity of residential wood heating is a public health concern [16]. And it will continue to grow should the supply and demand for this type of heating increase as extreme climatic events become more frequent and intense [1]. The aim of this study was to examine diverse perceptions and characteristics associated with wood heating through a survey carried out in 2005 in southern Québec, Canada [17] in the context of a research program aiming to propose climate change (CC) adaptation strategies that respect the environment as well as health and well-being.

## Methods

### Population studied and sample

**Figure 1:**
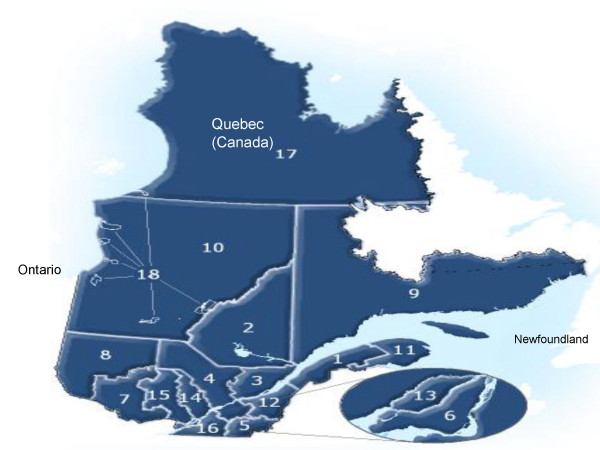
**Health regions of Québec (Canada)**. 1 : Bas-Saint-Laurent ; 2 : Saguenay-Lac-Saint-Jean ; 3 : Québec ; 4 : Mauricie-Centre-du-Québec; 5 : Estrie; 6 : Montréal; 7 : Outaouais; 8 : Abitibi-Témiscamingue; 9 : Côte-Nord; 11 : Gaspésie-Îles-de-la-Madeleine; 12 : Chaudière-Appalaches; 13 : Laval; 14 : Lanaudière; 15 : Laurentides; 16 : Montérégie. Source: MSSS, Service des Infocentres, 2006.

The population studied consisted of adults aged 18 years or older, resident of the Province of Québec south of the 49th parallel, namely all the health regions presented in Figure 1, except for regions 10, 17 and 18.

The sample was stratified by the health region of residence, and post-stratified by gender (in order to take into account the greater difficulty in reaching men [18]) (Table 1). Due to operational and budgetary constraints, we used random household sampling rather than within-household sampling. The respondents were contacted by a polling firm from random digit dialing of published residential telephone numbers. Confidential numbers were not used for ethical considerations. The study obtained ethical approval from Laval University's *Comité d'éthique de la recherche avec des êtres humains*. The consent was implicit as only adults whose phone number was published were interviewed; Laval University's ethics committee does not request any formal consent for such phone surveys.

**Table 1:** Stratification process by data collection

Name of the health regions of residence (number of the region)	Men ≥ 18 years^1^	Proportion	Men: n^2^	Women ≥ 18 years^1^	proportion	Women: n^2^	Men and women: n
Bas-Saint-Laurent (01)	77 455	0,014	36	82 070	0,015	38	74
Saguenay-Lac-Saint-Jean (02)	106 700	0,019	48	110 230	0,020	51	99
Capitale-Nationale (03)	246 320	0,044	112	271 710	0,048	122	234
Mauricie et Centre-du-Québec (04)	181 185	0,032	81	193 805	0,034	86	167
Estrie (05)	107 805	0,019	48	114 940	0,020	51	99
Montréal (06)	690 890	0,122	310	775 560	0,137	348	658
Outaouais (07)	116 260	0,021	53	124 345	0,022	56	109
Abitibi-Témiscaminque (08)	54 720	0,010	25	55 435	0,010	25	50
Côte-Nord (09)	37 710	0,007	18	36 900	0,007	18	36
Gaspésie-Îles-de-la-Madeleine (11)	37 635	0,007	18	39 550	0,007	18	36
Chaudière-Appalaches (12)	146 365	0,026	66	151 090	0,027	69	135
Laval (13)	127 755	0,023	58	138 870	0,025	64	122
Lanaudière (14)	144 030	0,026	66	148 550	0,026	66	132
Laurentides (15)	171 535	0,030	76	178 430	0,032	81	157
Montérégie (16)	474 705	0,084	213	505 235	0,090	229	442

TOTAL	2, 721, 070		1228	2, 926, 720		1322	2550

^1^Institut de la statistique du Québec (20).

^2^Thompson (21).

The sample was calculated using 2001 survey data [19], for a 95% confidence level and a precision level of 1.5%, for a 4-point Likert-type scale including 6 items [20]. The total sample was 5,088 respondents: half of them were contacted in the spring of 2005 (n = 2,543) on heat-related adaptation measures [21], and the other half during the following autumn (n = 2,545) on cold-related adaptation measures [17]. The present article pertains to the autumn data collection, in which 70.2% of the eligible people (n = 3,726) completed the questionnaire; 4.9% were not interviewed because data collection ended before the date of the appointment made with the polling firm; 6.6% could not be reached (e.g., answering machine); less than one percent (n = 7) did not complete the interview; and 18.2% refused to answer the study. The percentage of respondents and non-respondents were similar across health regions (p = 0.4).

### Data collection method

The polling firm collected individual responses by telephone (average duration: 20 minutes), seven days a week, from 9:30 a.m. to 9:30 p.m., using a computer system that allowed the order of the questions (essentially closed) to be randomly redistributed. More precisely, collection (from 15-09-2005 to 25-10-2005) allowed information to be gathered on behaviours adopted during a period of intense cold, socio-demographic characteristics, health status, dwelling, region of residence, the use of an automobile and a remote starter during the winter, consultation of weather reports, as well as on various perceptions and beliefs relating to climate change.

The questionnaire was developed according to the following six steps: 1/identifying the important issues to consider in the exploratory interviews [22] based on the literature on health and climate change; 2/conducting 21 face-to-face interviews (average duration: two hours), mainly to verify the understanding of some terms, identify the items to be retained as well as the sensitive issues to be excluded; 3/development of an initial version of the questionnaire; 4/conducting telephone interviews with 61 people aged 18 years or older (on average, four people per health region studied) to validate the clarity and precision of the questions, to comment on the questionnaire and to shorten it; 5/validation of the content of the questionnaire (French and English versions) by five experts working in the field of health and climate change in Canada; 6/conducting a qualitative pretest (n = 50) (two versions of the questionnaire) by the polling firm, at the start of each data collection.

### Analyses

The collected information was calibration weighted for the respondents' age and language, on the basis of 2001 census data [19]. Coefficients of variation (CV) were calculated (CV ≤ 15%: sufficiently precise estimates; CV between 15% and 25%: acceptable precision, estimates to be carefully interpreted; CV > 25%: low precision, estimates to be interpreted with circumspection) [23]. The percentage totals for a given variable may not be exactly 100%, due to rounding to the closest decimal (To simplify the presentation, percentages below 2% for missing data have not been reported). The analyses took into account the sample scheme stratified according to the health regions [24,25]. Wood heating was related to the independent variables using the Rao-Scott likelihood ratio chi-square test, which is a design-adjusted version of the Pearson chi-square test. The multivariate analyses were done using a logistic regression model with a stepwise method. The significance level required to be retained by the model: 0.2; to stay in the model: 0.1). The c index (area under the ROC curve; expected value = 0.5 to 1.0) [26] was used as an indicator of the discriminant capacity of the final multivariate statistical model. Finally, the presence of collinearity between the independent variables was checked (VIF > 10; condition > 30) [27].

## Results

### Characteristics of the respondents

Women, as well as people 35 to 64 years of age accounted for slightly more than half of the sample (Table 2). At least two participants out of three lived in a house and spoke only French (Table 2), except in Montréal and Laval (Table 3).

**Table 2:** Sociodemographic characteristics of the respondents: percentages corrected for stratified sampling and coefficients of variation

Variables		%^1^	CV^2^
Gender	Women	51.6	0.02
	Men	48.3	0.02
Age	18 to 34 years	29.1	0.03
	35 to 64 years	54.6	0.02
	65 years or more	16.2	0.05
First language learned at home	French only	81.0	0.01
	English only	6.1	0.09
	Language other than French or English	10.1	0.15
	English or French plus another language	2.9	0.08
Status of activities (last 12 months)	Employed	67.0	0.02
	Unemployed	8.4	0.07
	Student	3.4	0.15
	Retired	21.8	0.04
Income (before tax/from all sources/last 12 months)	Less than $ 15 000	9.3	0.07
	Between $ 15 000 and $ 29 999	17.2	0.05
	Between $ 30 000 and $ 44 999	17.8	0.05
	Between $ 45 000 and $ 59 999	14.1	0.05
	$ 60 000 or more	26.2	0.03
	Undisclosed^3^	15.2	0.05
Lives alone	Yes	18.2	0.04
	No	81.8	0.01
Region of residence	Eastern Québec	5.7	0.02
	Northern part of southern Québec	5.9	0.02
	Québec City region	14.6	0.01
	Centre of the province	6.4	0.02
	South of Montréal	21.1	0.01
	North of Montréal	15.7	0.01
	Montréal and Laval	30.8	0.01
Type of dwelling	House	64.9	0.01
	Apartment: ≤ 4 storeys	31.1	0.03
	Apartment > 4 storeys	3.9	0.11

^1^%: percentages. The total percentages for a given variable may not be exactly 100%, due to rounding to the closest decimal. To simplify the presentation, percentages below 2% for missing data have not been reported.

^2^CV, coefficients of variation. CV ≤ 15%: sufficiently precise estimates; CV between 15% and 25%: acceptable precision, estimates to be carefully interpreted; CV > 25%: low precision, estimates to be interpreted with circumspection [25].

^3^These participants, compared to those who disclosed their income strata, were more often women, individuals at least 65 years of age, and retired people.

**Table 3:** Some characteristics of the respondents by region of residence: percentages corrected for stratified sampling

	Region of residence						
Variables	Eastern Québec	Northern part of southern Québec	Centre of the province	Québec City region	South of Montréal	North of Montréal	Montréal and Laval
Type of dwelling:							
• House	87.4%^1^	78.8%	76.0%	67.2%	73.8%	85.0%	38.4%
• Apartment	12.6%	21.2%	24.0%	32.9%	26.3%	15.0%	61.6%
First language learned at home:							
• French only	96.0%	95.0%	96.2%	93.4%	86.1%	85.3%	60.8%
• other than French only	4.0%	5.0%	3.8%	6.6%	13.9%	14.7%	39.2%
Region of residence perceived as conducive to cold waves:							
• a lot	27.0%	41.5%	23.3%	31.9%	34.6%	33.9%	41.1%
• Average	44.6%	39.9%	55.1%	49.6%	46.9%	46.8%	40.8%
• not much	19.3%	17.1%	16.0%	16.0%	15.4%	15.0%	14.1%
• not at all	9.2%	1.6%	5.6%	2.5%	3.2%	4.3%	4.0%
Region of residence perceived as conducive to winter smog:							
• a lot	5.4%	0.6%	1.2%	3.3%	6.6%	4.4%	15.4%
• Average	12.4%	8.8%	19.2%	17.2%	22.5%	20.3%	31.5%
• not much	21.9%	21.4%	28.7%	33.5%	29.1%	29.4%	29.6%
• not at all	60.3%	69.3%	50.9%	46.1%	41.8%	45.9%	23.5%
Wood heating:							
• Yes	35.0%	34.8%	30.7%	23.0%	21.9%	24.9%	4.0%
• No	65.0%	65.2%	69.3%	77.0%	78.1%	75.2%	96.0%
Prohibition of wood heating when there is winter smog:							
• completely agree	26.2%	33.8%	29.8%	27.5%	36.1%	37.3%	48.5%
• do not completely agree	73.8%	66.2%	70.2%	72.5%	63.9%	62.7%	51.5%
Belief of the contribution of anthropogenic causes to climate change in the last fifty years:							
• average or a lot	79.3%	76.9%	82.8%	78.0%	85.2%	85.0%	84.1%
• not much or not at all	20.7%	23.1%	17.2%	22.0%	14.8%	15.0%	15.9%

^1^The total percentages for a given variable may not be exactly 100%, due to rounding to the closest decimal.

To simplify the presentation, percentages below 2% for missing data have not been reported.

More than three out of four respondents had access to a single source of energy at home, as follows: 60.8%, electricity; 8.0%, oil; 3.8%, natural gas or propane; 3.7%, firewood. The other participants (22.2%) combined some of these sources (e.g., oil, gas, wood), with three out of five (59.5%) combining electricity and wood.

### Factors associated with residential wood heating

During the winter, 18.5% of the respondents heated with wood occasionally or daily and more precisely: 1.7%, less than once a week; 4.5%, a few days a week but not every day; and 11.9%, every day.

Respondents with higher incomes used wood as a primary or secondary source of energy in a higher proportion than the other participants, as well as the respondents aged between 35 to 64 years, who spoke French only or in addition to another language, or who lived with children or with other people (Table 4).

**Table 4:** Use of residential wood heating in southern Québec for various respondents characteristics: percentages corrected for stratified sampling and p value

	Wood heating		p value^1^
Variables	yes	no	
*Sociodemographic characteristics*			
• Gender:			0.1885
• men	20.3%^2^	79,7%	
• women	18.2%	81.8%	
Age:			0.0004
• 18–34 years	16.2%	83.8%	
• 35–64 years	22.4%	77.6%	
• 65 years or more	14.5%	85.5%	
Status of activities (last 12 months):			0.0374
• employed	20.3%	79.7%	
• unemployed	21.8%	78.2%	
• student	11.6%	88.4%	
• retired	15.7%	84.3%	
Income before tax, from all sources (last 12 months):			< 0.0001
• < $45,000	16.8%	83.2%	
• ≥ $45,000	23.6%	76.4%	
• not disclosed	14.4%	85.6%	
First language learned at home:			< 0.0001
• French only	21.9%	78.2%	
• English only	4.9%	95.1%	
• other language in addition to French or English	20.6%	79.4%	
• language other than French and English	6.1%	93.9%	
Status as parent:			< 0.0001
• no children	14.7%	85.3%	
• adult children only	20.0%	80.0%	
• at least one minor child	23.2%	76.8%	
Cohabitation:			< 0.0001
• lives with other people (related or not)	21.0%	79.0%	
• lives alone	11.9%	88.1%	
*Health status*			
Perceived health status:			0.1624
• very good	20.1%	79.9%	
• good	19.8%	80.2%	
• average	15.6%	84.4%	
• bad	12.8%	87.2%	
Having at least one chronic disease diagnosed by a physician and having had it for at least six months			0.0809
• yes	16.9%	83.1%	
• no	20.0%	80.0%	
Observance of behaviours according to the preventive advice issued by health professionals			0.5748
• always	19.3%	80.7%	
• often	18.3%	81.7%	
• sometimes	21.8%	78.2%	
• rarely	17.6%	82.4%	
• never	19.4%	80.6%	
Perceived influence of extreme meteorological conditions (e.g., heat waves) on health:			0.0179
• a lot	15.2%	84.8%	
• average	15.4%	84.6%	
• not much	18.0%	82.0%	
• not at all	21.2%	78.8%	
*Dwelling*			
Type of dwelling:			< 0.0001
• house	28.1%	71.9%	
• apartment	2.6%	97.4%	
Perceived efficiency of the dwelling's insulation against moisture:			< 0.0001
• very good	25.2%	74.8%	
• good	18.9%	81.1%	
• average	15.6%	84.5%	
• poor	7.3%	92.7%	
Perceived efficiency of the dwelling's insulation against cold:			< 0.0001
• very good	24.5%	75.6%	
• good	20.4%	79.6%	
• average	12.8%	87.2%	
• poor	6.3%	93.8%	
Perceived efficiency of the dwelling's insulation against heat:			< 0.0001
• very good	27.1%	72.9%	
• good	19.9%	80.2%	
average	13.6%	86.4%	
• poor	3.7%	96.3%	
Addition of insulating materials since the dwelling was built:			< 0.0001
• yes	26.6%	73.4%	
• no	18.1%	81.9%	
• don't know	6.4%	93.6%	
Replacement of doors or windows since the dwelling was built:			0.3479
• yes	20.5%	79.5%	
• no	18.9%	81.1%	
Dwelling built before 1983^3^:			0.1684
• yes	18.7%	81.3%	
• no	21.5%	78.5%	
• unknown^4^	17.4%	82.6%	
*Region of residence*			
Region lived in:			< 0.0001
• Eastern Québec	35.0%	65.0%	
• Northern part of southern Québec	34.8%	65.2%	
• Central Québec	30.7%	69.3%	
• Québec City region	23.0%	77.0%	
• North of Montréal	24.9%	75.2%	
• South of Montréal	21.9%	78.1%	
• Montréal and Laval	4.0%	96.0%	
Region of residence perceived as conducive to ice storms			0.0828
• a lot	15.2%	84.8%	
• average	19.6%	80.4%	
• not much	20.1%	79.9%	
• not at all	22.8%	77.2%	
Region of residence perceived as conducive to winter smog			0.0002
• a lot	11.6%	88.4%	
• average	15.8%	84.2%	
• not much	20.2%	79.8%	
• not at all	23.3%	76.7%	
Region of residence perceived as conducive to cold waves			0.0003
• a lot	14.0%	86.0%	
• average	21.8%	78.2%	
• not much	24.3%	75.7%	
• not at all	23.6%	76.4%	
*Transport*			
Frequency of use of an automobile:			< 0.0001
• daily	21.9%	78.1%	
• occasionally	21.5%	78.5%	
• never	6.8%	93.2%	
Use of a remote starter in winter			0.2007
• yes	23.4%	76.6%	
• no	20.8%	66.1%	
*Consultation of meteorological information in the media*			
• Temperature:			0.2666
• always	20.2%	79.8%	
• often	19.2%	80.8%	
• sometimes	19.5%	80.5%	
• rarely	17.7%	82.3%	
• never	12.5%	87.5%	
Smog warning:			0.1205
• always	17.3%	82.7%	
• often	16.4%	83.6%	
• sometimes	19.2%	80.8%	
• rarely	23.3%	76.7%	
• never	20.1%	79.9%	
Intense cold warning:			0.0594
• always	19.8%	80.2%	
• often	19.9%	80.1%	
• sometimes	21.9%	78.1%	
• rarely	15.4%	84.6%	
• never	13.6%	86.4%	
Belief of the contribution of anthropogenic causes to climate change in the last fifty years:			0.1904
• a lot	19.4%	80.6%	
• average	18.5%	81.5%	
• not much	22.7%	77.3%	
• not at all	15.6%	84.4%	
Prohibition of wood heating when there is winter smog:			< 0.0001
• completely agree	10.4%	89.6%	
• somewhat agree	17.5%	82.5%	
• somewhat disagree	24.2%	75.8%	
• completely disagree	38.5%	61.5%	

^1^Wood heating was related to the independent variables using the Rao-Scott likelihood ratio chi-square test, which is a design-adjusted version of the Pearson chi-square test.

^2^The total percentages for a given variable may not be exactly 100%, due to rounding to the closest decimal. To simplify the presentation, percentages below 2% for missing data have not been reported.

^3^In 1983, the Law on Conservation of energy in buildings was adopted in Québec to insure a minimal performance of the thermal insulation in walls and ceilings.

^4^Among these respondents (< 5% of the participants), 73,3% lived in apartments.

Higher percentages of respondents heating with wood at least occasionally during the winter were observed for those individuals living a) in a house, b) in a dwelling built in 1983 or after, c) to which insulating materials had been added since its construction or in which the insulation efficiency was considered appropriate as protection against heat, cold and humidity (Table 4).

The prevalence of wood heating was higher in the peripheral regions than in the more urban regions located within the study area (Table 4), in particular in populated urban environments such as Montreal (Table 3). Similarly, higher percentages of respondents heating with wood at least occasionally during the winter were observed for those individuals who considered their region of residence to be at lesser risk of experiencing winter smog, or for those participants who believed in the contribution of anthropogenic causes to climate change in the last fifty years (Table 4).

The prevalence of residential wood heating was higher with participants who rarely or never consulted the smog warning in the media compared to those consulting more, or with participants who completely disagreed with the prohibition of wood heating during smog episodes in winter, as compared to respondents who somewhat disagreed, agreed somewhat, or completely with the adoption of this strategy (Table 4).

In the multivariate analysis, ten of the variables associated with the use of residential wood heating seemed to differentiate occasional and daily users from non-users, and these are : (1) to live in a peripheral region; (2) to live in a house; (3) to not completely agree with the prohibition of wood heating when there is smog in winter; (4) to live in a dwelling built in 1983 or later, (5) to live in a dwelling to which insulating materials had been added since its construction, (6) or in which the insulation efficiency was considered appropriate as protection against warm conditions; (7) to consult smog warnings in the media; (8) to believe in the contribution of anthropogenic causes to climate change in the last fifty years; (9) to have income of at least 45 000 $; and (10) to have first learned at home, French only or in addition to another language. Among the 2^10-1 ^(or 1023) sub-models, 64 models had a c index (area under the ROC curve; expected value = 0.5 to 1.0) over 0.8. The most discriminant model (c index: 0.8176) included nine of the preceding variables (except the addition of insulating materials) and the most economic model, with a similar discriminant capacity (c index: 0.8029), had only the first three (Table 5).

**Table 5:** Indicators differentiating occasional or daily users of residential wood heating from non-users: multivariate analysis corrected for stratified sampling

Variables	OR^1^	CI_95%_^1^	P value^2^	c index^3^	Rank
Model1				0.8176	1^4^
*Sociodemographic characteristics*					
Income before tax, from all sources (last 12 months):			0.0056		
• < $45,000	reference group				
• ≥ $45,000	1.1	0.9;1.5			
• not disclosed	0.6	0.4 ; 0.9			
First language learned at home:			0.0257		
• French only	reference group				
• English only	0.3	0.1 ; 0.7			
• other language in addition to French or English	1.0	0.4 ; 2.8			
• languages other than French and English	0.6	0.3 ; 1.6			
*Dwelling*					
Type of dwelling:			< 0.0001		
• apartment	reference group				
• house	10.8	6.7 ; 17.4			
Perceived efficiency of the dwelling's insulation against heat:			0.0009		
• very good	reference group				
• good	0.7	0.5 ; 0.9			
• average	0,6	0,4 ; 0.8			
• poor	0.3	0.1 ; 0.6			
Dwelling built before 1983^1^:			0.0159		
• yes	reference group				
• no	3.1	1.4 ; 6.7			
• unknown	1.0	0.8 ; 1.3			
*Region of residence*					
Region lived in:			< 0.0001		
• Eastern Québec	reference group				
• Northern part of southern Québec	1.2	0.7 ; 2.1			
• Central Québec	1.0	0.6 ; 1.7			
• Québec City region	0.7	0.4 ; 1.1			
• North of Montréal	0.7	0.5 ; 1.1			
• South of Montréal	0.7	0.5 ; 1.1			
• Montréal and Laval	0.2	0.1 ; 0.4			
*Consultation of smog warning in the media*			0.0112		
• always	reference group				
• often	0.9	0.6 ; 1.4			
• sometimes	1.2	0.8 ; 1.7			
• rarely	1.4	0.9 ; 2.1			
• never	0.8	0.5 ; 1.1			
*Beliefs*					
Belief of the contribution of anthropogenic causes to climate change in the last fifty years:			0.0388		
• a lot	reference group				
• average	0.8	0.6 ; 1.1			
• not much	1.0	0.7 ; 1.5			
• not at all	0.5	0.3 ; 0.9			
Prohibition of wood heating when there is winter smog:			< 0.0001		
• completely agree	reference group				
• somewhat agree	1.8	1.3 ; 2.6			
• somewhat disagree	2.6	1.9 ; 3.6			
• completely disagree	5.2	3.6 ; 7.5			

Model2				0.8029	64^5^
Type of dwelling:			< 0.0001		
• apartment	reference group				
• house	10.0	6.2 ; 16.2			
Region lived in:			< 0.0001		
• Eastern Québec	reference group				
• Northern part of southern Québec	1.1	0.7 ; 1.9			
• Central Québec	1.0	0.6 ; 1.7			
• Québec City region	0.7	0.4 ; 1.1			
• North of Montréal	0.7	0.4 ; 1.0			
• South of Montréal	0.7	0.4 ; 1.0			
• Montréal and Laval	0.2	0.1 ; 0.3			
Prohibition of wood heating when there is winter smog:			< 0.0001		
• completely agree	reference group				
• somewhat agree	1.8	1.3 ; 2.6			
• somewhat disagree	2.5	1.8 ; 3.4			
• completely disagree	4.4	3.1 ; 6.2			

^1^OR: odds ratio; IC_95%_: 95% confidence interval

^2^p value associated with the Wald test by means of logistic regression.

^3^Area under the ROC curve; between 0.8 and 0.9: good model. No collinearity between the independent variables was observed.

^4^64 models had a c index over 0.8. This model ranked first.

^5^64 models had a c index over 0.8. This model ranked last.

More specifically (Table 5, models 1 and 2), compared to the respondents living outside the large urban regions of the province of Québec (e.g. regions 2 or 9, Figure 1), the odds of wood heating was 10 times lower for participants living in the cities of Montréal or Laval. The odds of wood use for heating was 10 times higher for residents of a house than respondents living in an apartment, a high proportion of whom lived in the regions of Montréal and Laval (Table 3). And compared to the respondents strongly hoping that wood heating would be prohibited during the presence of smog in winter, the odds of wood use for heating was 1.8 times higher for participants somewhat agreeing with this solution reported using this type of supplementary heating. This odds ratio was at least 2 for the participants somewhat disagreeing with this solution and at least 4 for the respondents completely disagreeing.

## Discussion

This population survey on beliefs and adaptations about climate change, including residential wood heating, did not intend to measure the impact of wood burning on the levels of air pollutants, nor the impact of related home indoor pollutants on the health of its inhabitants. However, this survey found the prevalence of residential wood heating to be 18.5% in Quebec (11.9%, every day), which is very close to the approximately 20% documented by the 2003 Canadian Survey of Household Energy Use [28]. As well, heating with wood during the winter was not influenced by smog warnings. From a public health standpoint, these results are of concern for several reasons.

First, wood smoke associated with residential wood burning has known negative impacts on health. It is likely to cause a variety of adverse respiratory health effects, including increases in respiratory symptoms, lung function deterioration, and increased visits to emergency departments and hospitalizations [29]. Furthermore, wood smoke is an important contributor to particle concentrations [29] and its increased use could result in a substantial increase in the number of premature deaths [30]. Clearly, there seems to be no reason to assume that the effects of particulate matter in areas polluted by wood smoke are weaker than elsewhere [31].

Second, in 2003, 30% of the atmospheric emissions generated by the total of fixed sources in Québec were attributable to wood heating and are increasing [32]. It is likely that residential biomass combustion will become even more widespread, given the recent upward trend in the costs of oil and natural gas [29]. Moreover, the use of wood as a primary or secondary source of heat is presently encouraged by the Canadian government as a useful adaptation in defense against the harmful effects of prolonged power outages brought on by extreme climatic events[9]. Furthermore, a close and continuous monitoring of the evolution in residential wood heating does not exist at the present time.

Third, even in densely populated urban environments where most people live in apartments and where the prevalence of wood heating is very low (e.g. Montréal in this survey), air quality can be severely affected by wood smoke. For example, air quality measures implemented between 1999 and 2002 in Montréal have demonstrated that some atmospheric pollutants (e.g. particulate matter) in a residential district using wood heating to a great extent were up to five times higher in winter than in summer, and up to two times higher in winter in that district than in downtown high traffic areas [33].

Fourthly, this survey found that the use of residential wood heating does not seem to be influenced either by the perception of living in a region conducive to smog, or by the smog warnings emitted by Environment Canada through the media. This may be due to the fact that the Info-Smog program did not cover the regions with the highest prevalence of wood heating during this study. This program informs the population through the media about the presence of meteorological conditions conducive to increased atmospheric pollution, and sends, at the same time, advice about reducing the sources of pollution and their health impacts [34]. This is a possible but refutable hypothesis: the perception and warnings about smog do not seem to affect the use of an automobile or a remote starter (two other sources of smog) in Montréal [17], where Info-Smog has existed since its creation in 1994 [34]. However, many other determinants – besides the perception of risk and the knowledge relating to it – can promote the adoption of a health-related behaviour, and these are mainly habit, social determinants (e.g., behaviour standards, pressure felt), beliefs, moral principles [35,36], and other variables (e.g. type of dwelling, accessibility of wood) in particular in regions characterized by colder and longer winters [37].

Finally, the average age of wood stoves used as the primary heating system in Canada was 12 years in 2003 [38] and it is likely that the stoves used as a secondary source of heat are just as old. Chances are that a significant proportion of these appliances are not certified according to the standards of the United States Environmental Protection Agency (EPA) [39] or not approved by the Canadian Standards Association (environmental performance standard B.415.1-04), if only because the costs of purchasing and installing the new technologies would be between $1,800 and $5,000 per stove [40]. In addition, no Canadian law prohibits the sale of uncertified wood burning appliances, which emit in nine hours as much fine particulate matter into the atmosphere as a certified stove operating for 60 hours, or as an intermediate type automobile traveling 18 000 km in a year [7]. In this survey, the type of appliance and the year of acquisition were not evaluated. However, it would be surprising that these specifications differ greatly from the rest of Canada.

Consequently, in Canada and other similar cold regions (e.g. Northern Europe, Russia), it would be appropriate to implement long-term national programs on residential wood heating to reduce pollutant emissions at source. Such a program could simultaneously include feasible adaptation measures of the "no-regrets" type (which are measures with climatic and non-climatic benefits). Such an approach would include educational measures (e.g. observance of good practice), incentive measures (e.g. financial assistance for replacing a conventional appliance and its recycling), and legislative measures including various control strategies (e.g. prohibition of the sale of uncertified wood burning appliances, prohibition of wood heating on smog days) [40,41] plus simultaneous mechanisms to ensure their application (e.g., high fines for polluting citizens and municipal administrations). In addition, close and continuous monitoring [42] of the evolution in residential wood heating would be necessary, including variables related to atmospheric and indoor pollutants, appliances, their actual use, installation and maintenance, users, the natural environment (e.g., wind, topographical characteristics) and the dwellings (e.g., ventilation of the dwelling). Finally, research is needed on the cultural and psychosocial determinants of heating practices to help focus intervention programs and on the health impacts of wood heating for highly exposed groups under conditions of a developed country [30], as is the case for Québec.

## Conclusion

In recent years, much has been written about heat waves that have occurred in some industrialized countries. While this is important, it would be also desirable to remember that there will still be winters and periods of intense cold in the northern regions, such as Canada, and that people will still have to continue to adapt to them. It is indisputable that wood heating is an interesting adaptation strategy for protection against the cold during extreme climatic events that can lead to prolonged power outages, particularly when this renewable energy resource is easily accessible in several northern countries. However, in the light of the results of this study and the literature on air pollution and climate change, it is important to state that much remains to be done, individually and collectively, to avoid wood heating becoming in fact a *maladaptation*. In light of the precautionary principle, the current imprecise and incomplete "scientific evidence" associated to the health and environmental impacts of residential wood heating is, in our view, an additional reason to implement a long-term national program on improved and controlled wood heating as part of "no-regrets" adaptation measures to climate change that brings more heating autonomy to dwellings during severe climate events while reducing air pollution and its associated health impacts.

## Competing interests

The authors declare that they have no competing interests.

## Authors' contributions

DB lead the conception, design, analysis and interpretation of the study. DB and PG wrote the paper. PG, PV and BA reviewed the paper and were involved in the design of questionnaire and sampling. All gave their final approval of this version.

## Pre-publication history

The pre-publication history for this paper can be accessed here:


